# Author Correction: Natural selection and genetic diversity maintenance in a parasitic wasp during continuous biological control application

**DOI:** 10.1038/s41467-026-74366-5

**Published:** 2026-06-30

**Authors:** Bingyan Li, Yuange Duan, Zhenyong Du, Xuan Wang, Shanlin Liu, Zengbei Feng, Li Tian, Fan Song, Hailin Yang, Wanzhi Cai, Zhonglong Lin, Hu Li

**Affiliations:** 1 Department of Entomology and MOA Key Lab of Pest Monitoring and Green Management, College of Plant Protection, China Agricultural University, Beijing, 100193 China; 2 Tobacco Company, Yuxi, 653100 China; 3 Yunnan Tobacco Company of China National Tobacco Corporation, Kunming, 650011 China

Correction to: *Nature Communications* 10.1038/s41467-024-45631-2, published online 14 February 2024

In the version of this article initially published, there was an error in Fig. 5e where the red and blue colors of the bar plots (now appearing at left and right, respectively) were inadvertently reversed; the figure is now amended in the HTML and PDF versions of the article.

