# Supplementary material for: Natural selection and genetic diversity maintenance in a parasitic wasp during continuous biological control application
Source: Nat Commun. 2024 Feb 14;15:1379. doi: 10.1038/s41467-024-45631-2 (PMC10866907; doi:10.1038/s41467-024-45631-2)
Supplement: Supplementary file 1 — Supplementary Information [file 41467_2024_45631_MOESM1_ESM.pdf]

Natural selection and genetic diversity maintenance in a parasitic wasp during continuous biological control application

Li *et al.*

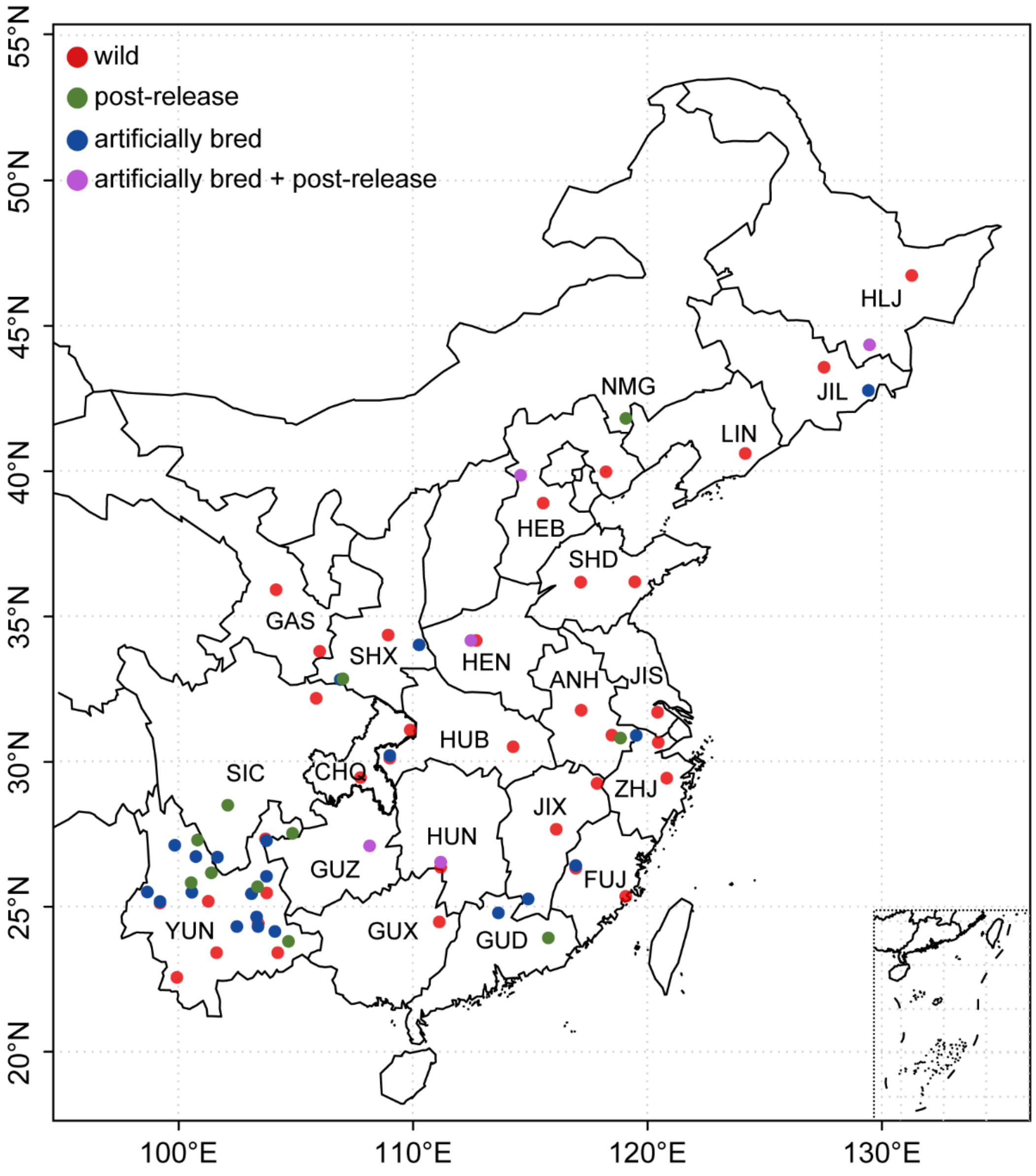

Supplementary Figure 1 Geographic locations of sampled populations. The wild (red), post-release populations (green) and artificially bred (blue) were indicated by different colors, and the purple point indicates the region from which we collected artificially bred and post-release populations. The map was drawn by the R Packages maps (https://cran.r-project.org/web/packages/maps). The detail information of each population abbreviations is listed in Supplementary Data 1 and Supplementary Data 2.

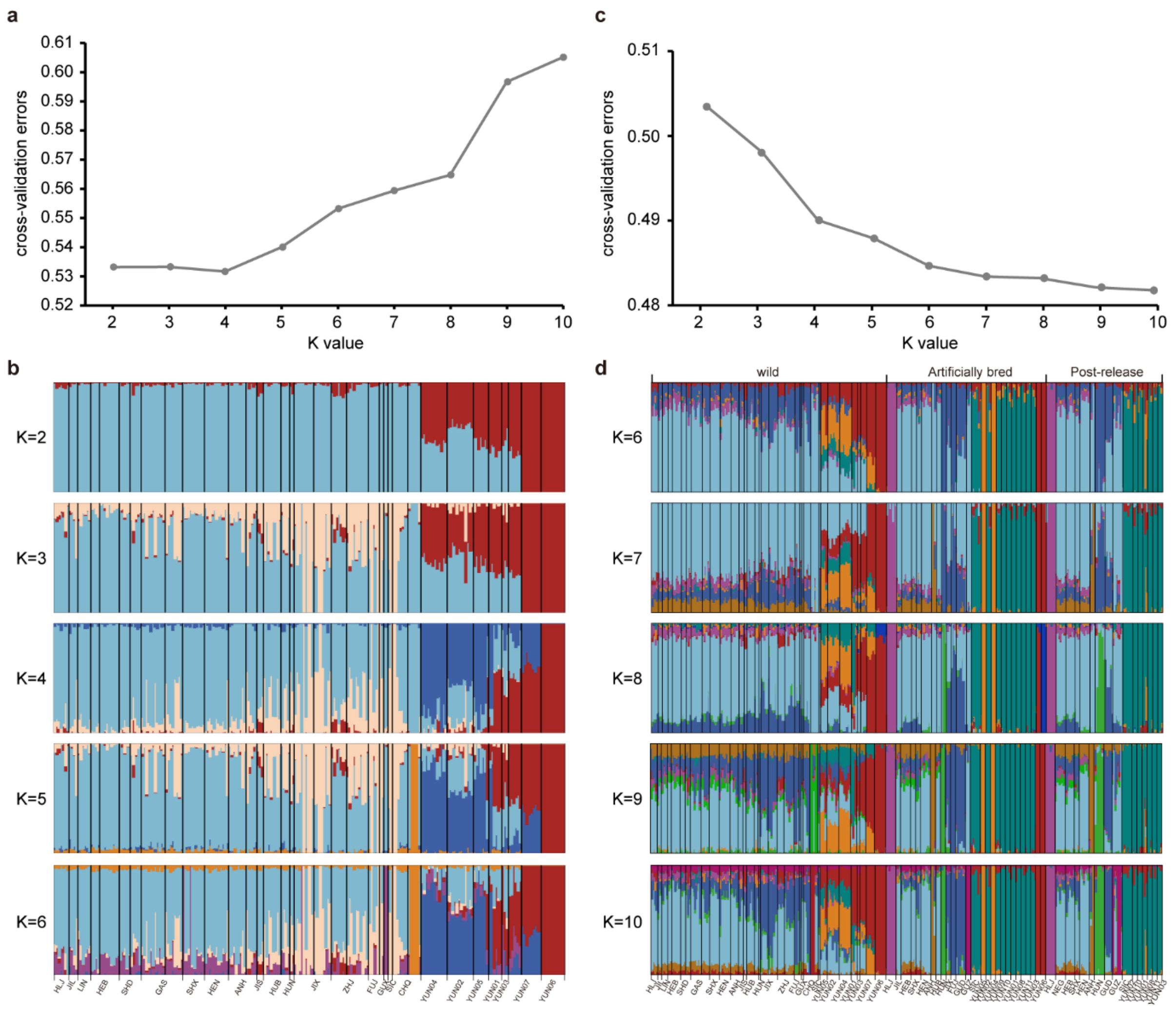

Supplementary Figure 2 ADMIXTURE cross-validation error and genetic structure of wild populations and all populations. a ADMIXTURE cross-validation error of wild populations for K value from 2 to 10. **b** genetic structure of wild populations when K = 2 to 6. **c** ADMIXTURE cross-validation error of all populations for K value from 2 to 10. **d** genetic structure of all populations when K = 6 to 10. Source data for Supplementary Figure 2a and 2c are provided as a Source Data file.

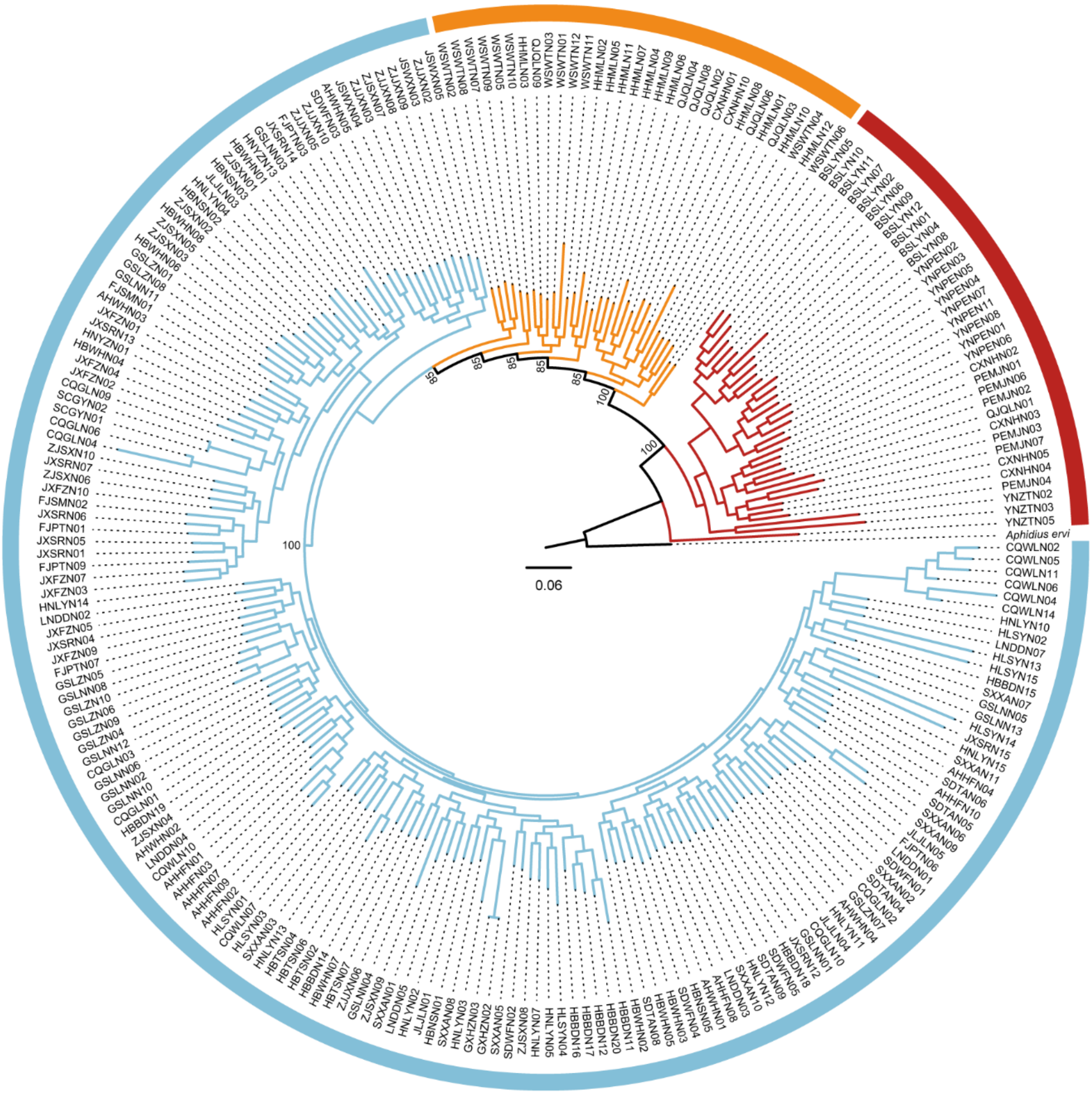

Supplementary Figure 3 Maximum-likelihood (ML) tree of wild individuals built by RAxML with 1,000 bootstraps. The tree with red and orange line indicated individuals from Yunnan Province, while the bule line indicated individuals from other regions expect Yunnan. Outgroup species *A. ervi* was added to the root of the tree.

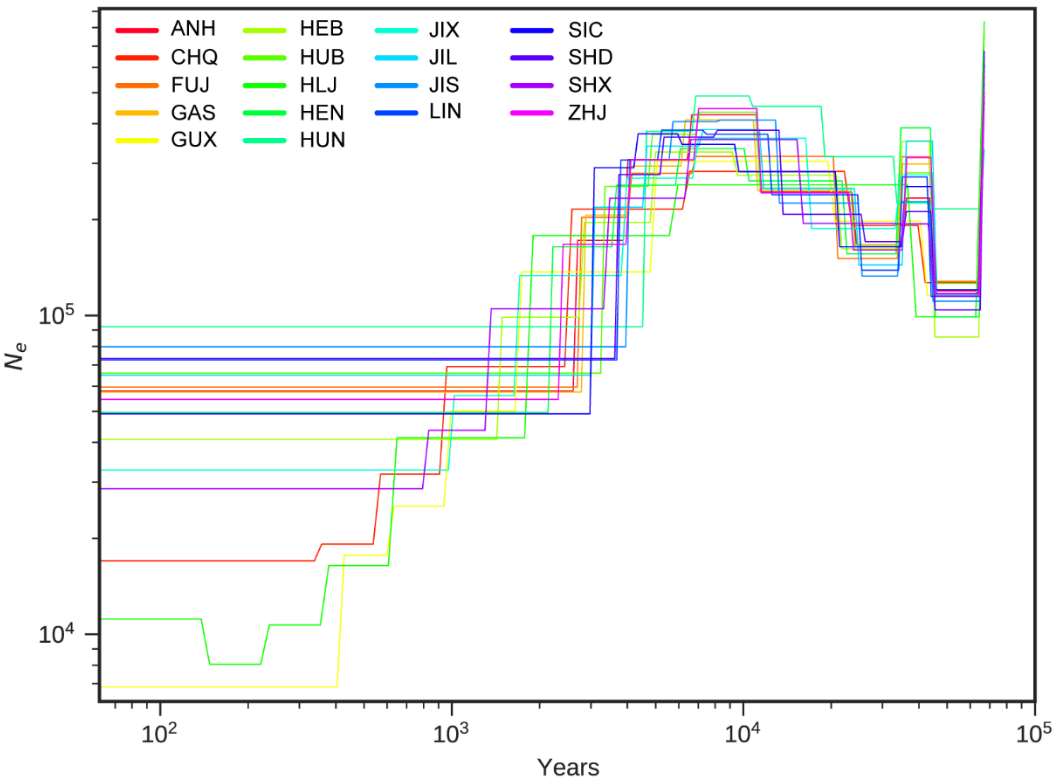

Supplementary Figure 4 Demographic history of wild populations from each region out of Yunnan Province predicted by SMC++. The detail information of each population abbreviations is listed in Supplementary Data 1 and Supplementary Data 2. Source data are provided as a Source Data file.

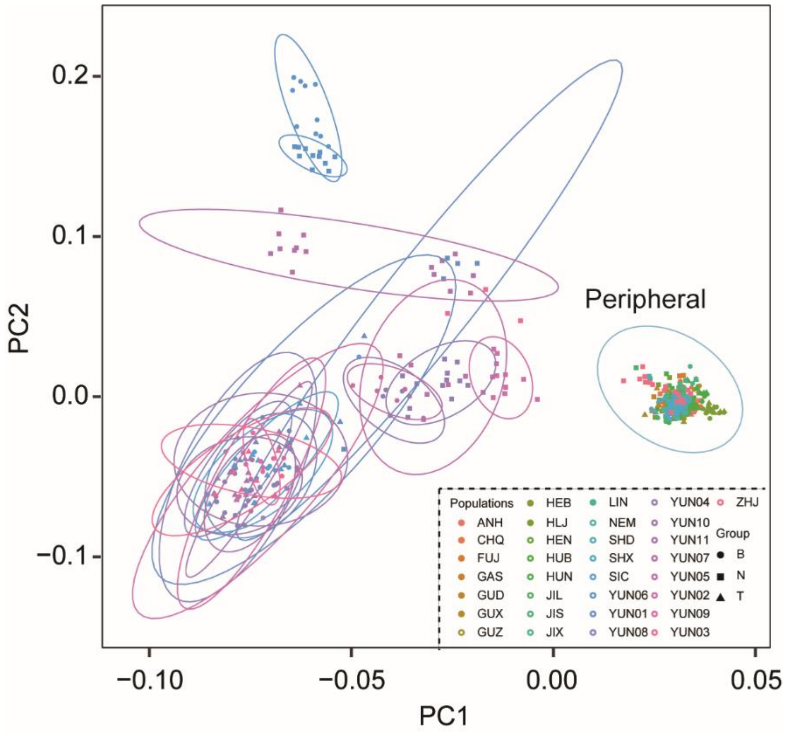

Supplementary Figure 5 PCA analysis of all samples, including wild, artificially bred and post-release populations. B: artificially bred populations; N: wild populations; T: post-release populations. The detail information of each population abbreviations is listed in Supplementary Data 1 and Supplementary Data 2. Source data are provided as a Source Data file.

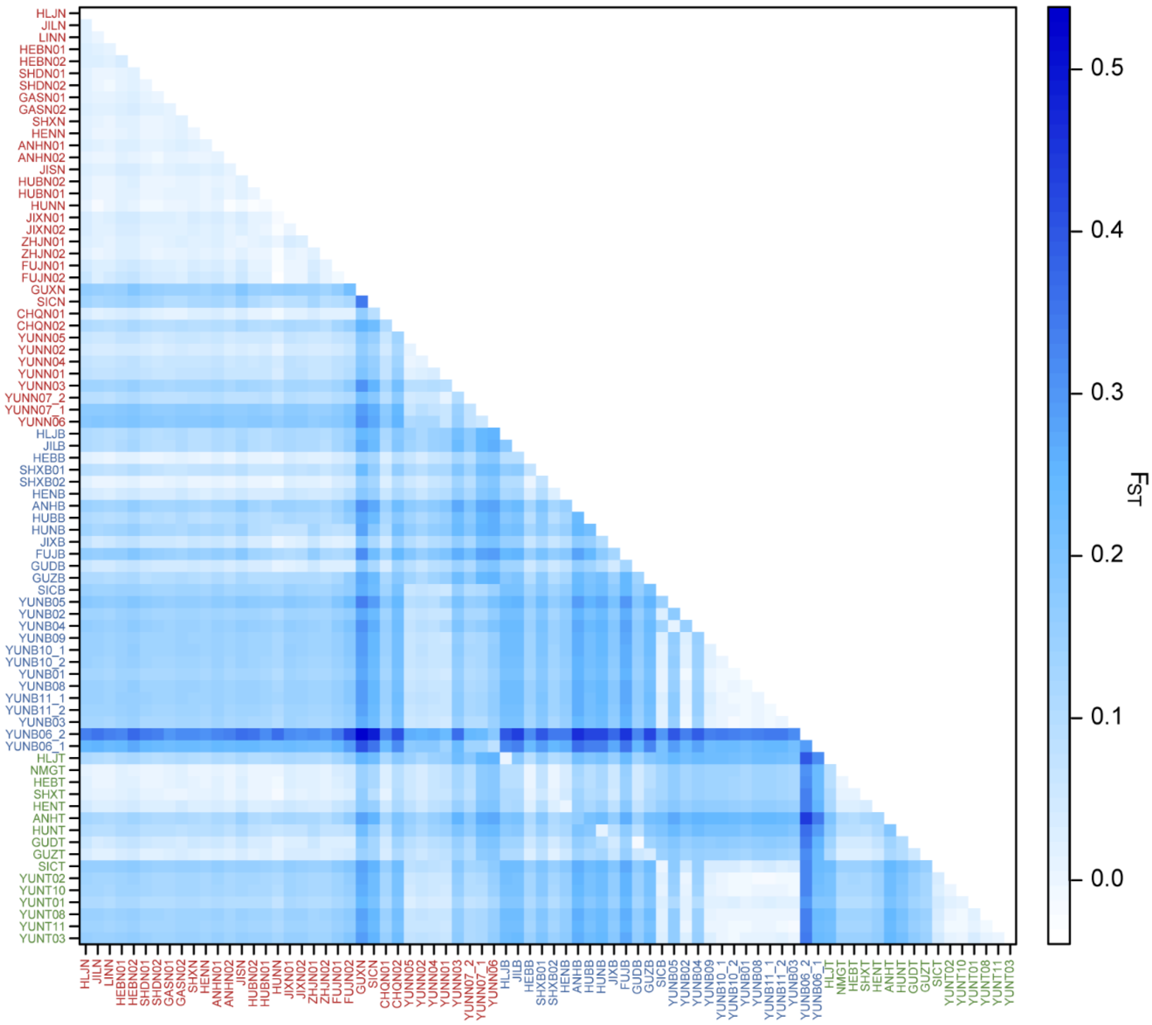

Supplementary Figure 6 Genetic differentiation of both population pairs. Populations ID in red, green and blue indicated wild, post-release and artificially bred populations, respectively. The detail information of each population abbreviations is listed in Supplementary Data 1 and Supplementary Data 2. Source data are provided as a Source Data file.

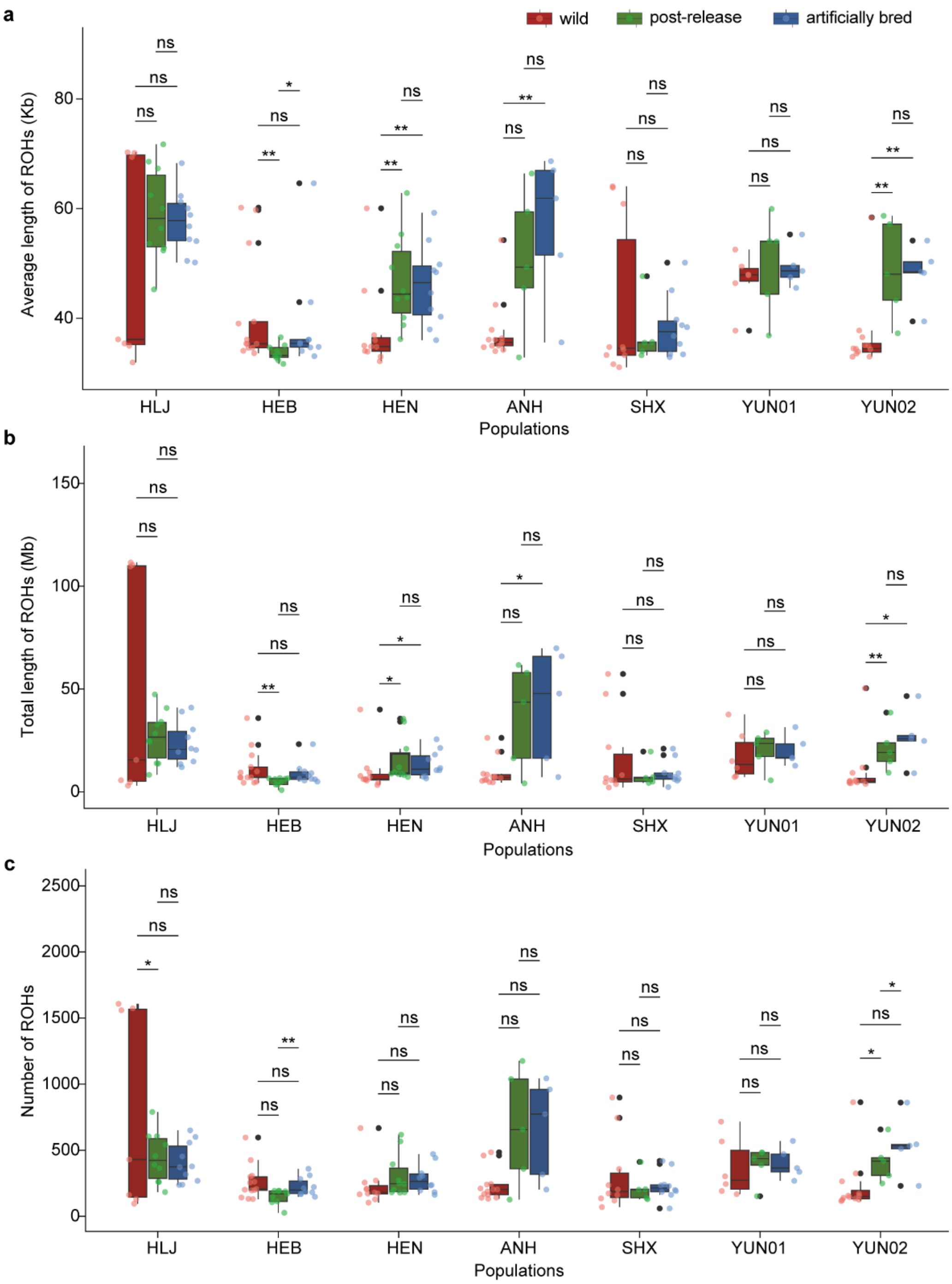

Supplementary Figure 7 Runs of homozygosity (ROH) across sampling locations. a Average ROH segment size. **b** Total length of ROH segments. **c** Number of ROH segments. Boxes in red, green and blue indicated wild, post-release and artificially bred populations from each region, respectively. The sample size of each population from per province was list in Supplementary Data 1 and Supplementary Data 2. In all boxplots, values for each of the individuals are shown as points surrounding boxplots. Boxplots depict the median, 25% and 75% quartiles. *P* values were obtained using two-sided Wilcoxon rank sum tests and adjusted via the Bonferroni test. *, $P < 0.05$; **, $P < 0.01$; ns, non-significant difference. Source data are provided as a Source Data file.

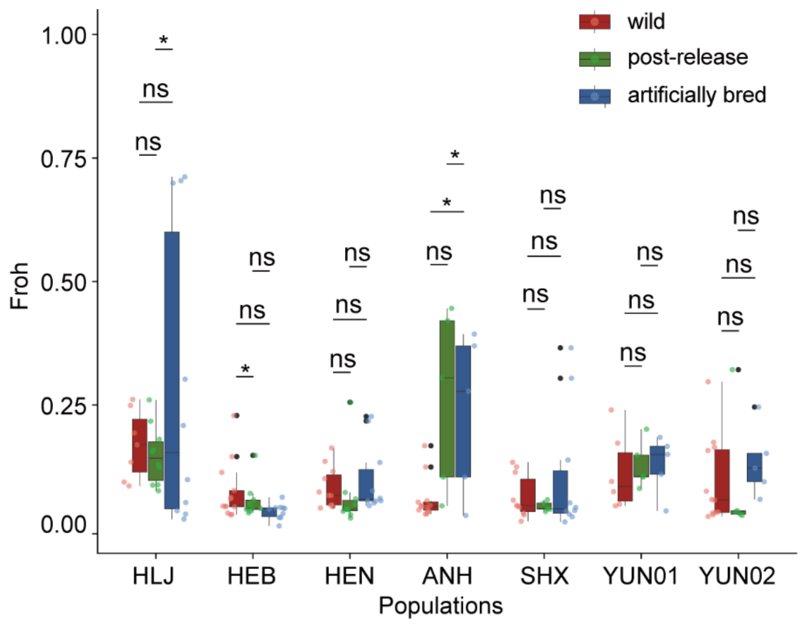

Supplementary Figure 8 Inbreeding level of populations from different regions measured by runs of homozygosity. Boxes in red, green and blue indicated wild, post-release and artificially bred populations from each region, respectively. The sample size of each population from per province was list in Supplementary Data 1 and Supplementary Data 2. Values for each of the individuals are shown as points surrounding boxplots. Boxplots depict the median, 25% and 75% quartiles. P values were obtained using two-sided Wilcoxon rank sum tests and adjusted via the Bonferroni test. ns indicated non-significant difference. *, $P < 0.05$. Source data are provided as a Source Data file.

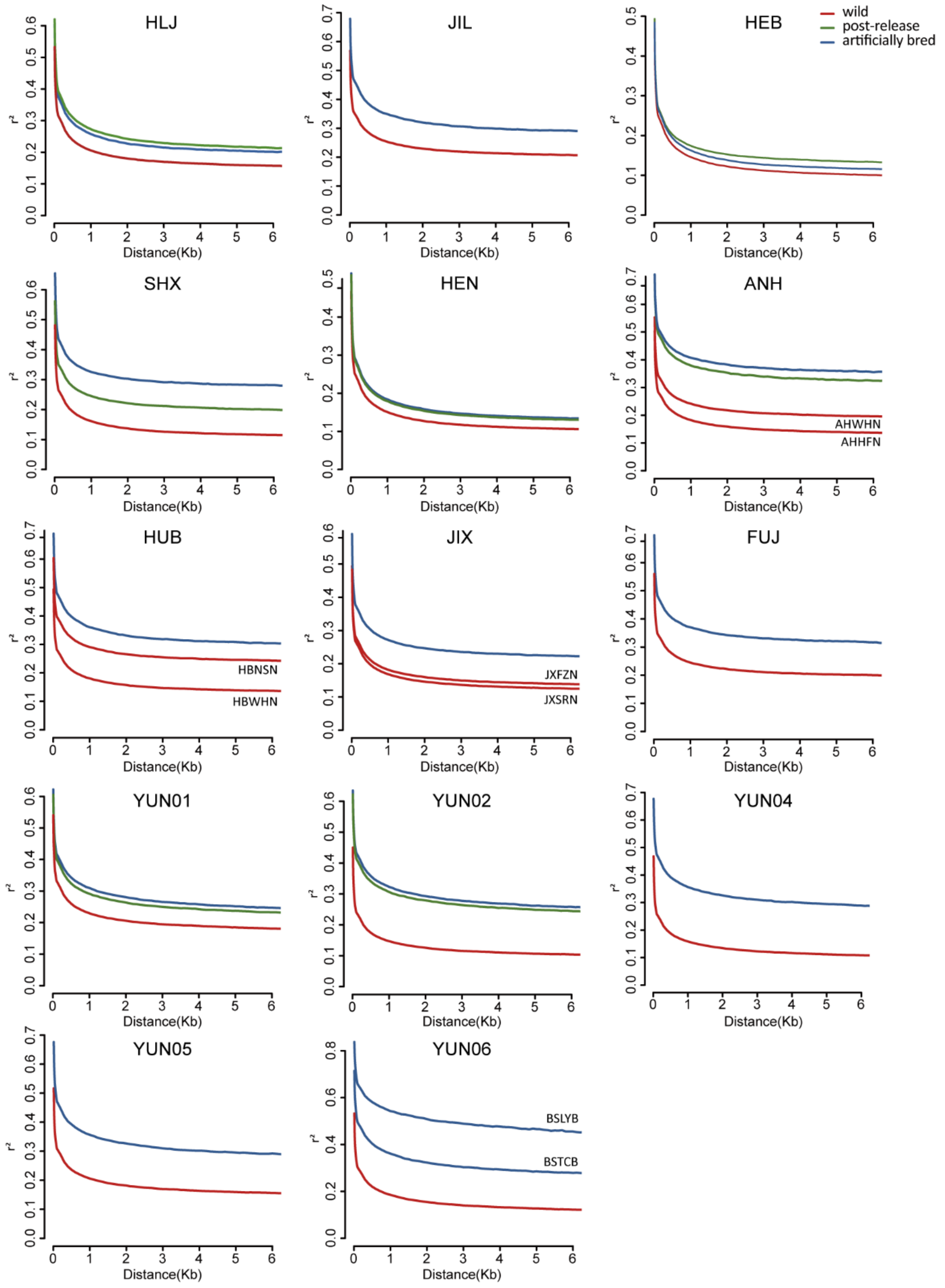

Supplementary Figure 9 Decay of linkage disequilibrium of artificially bred (blue), post-release (green) and wild populations (red) from each region. Source data are provided as a Source Data file.

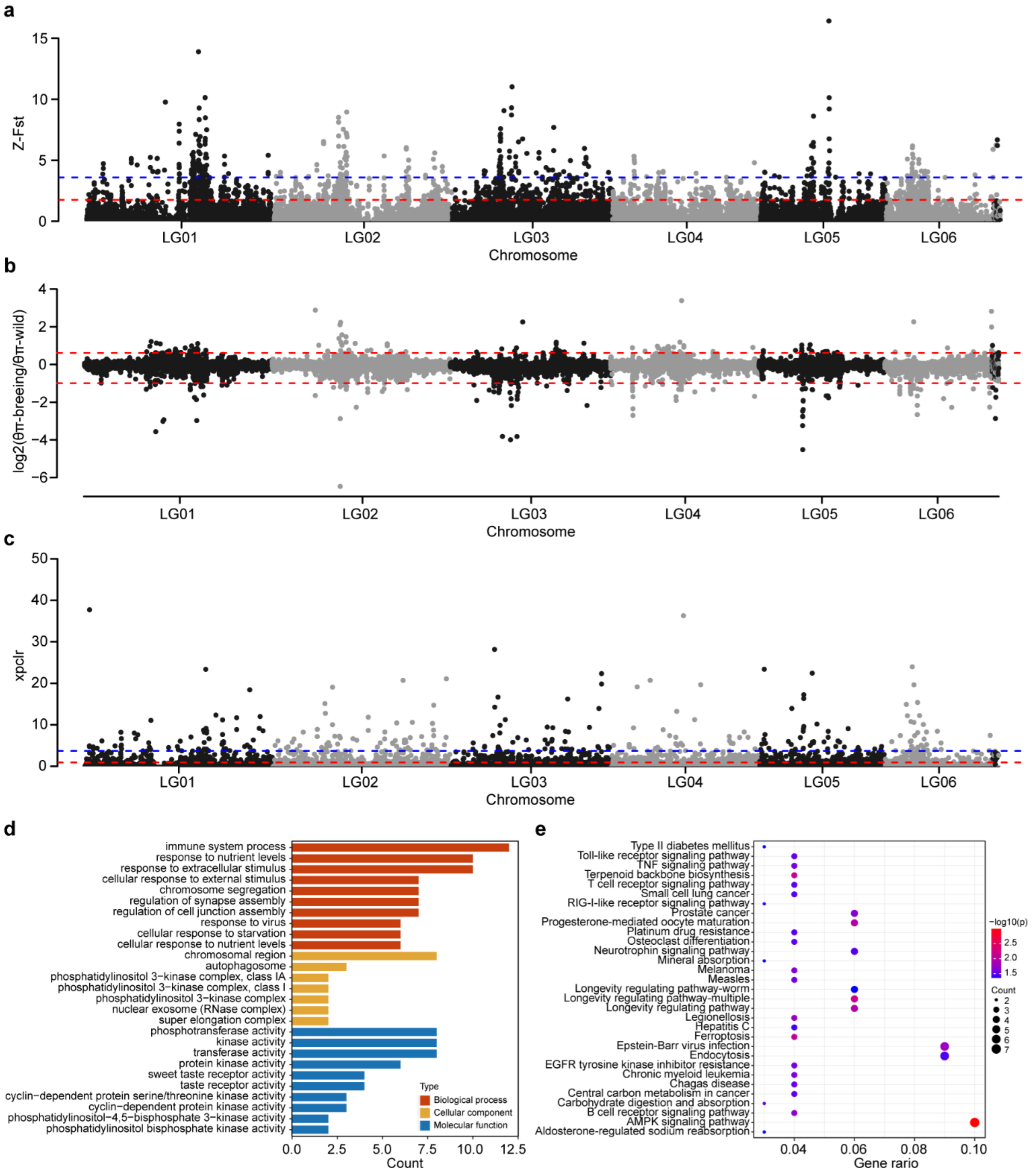

Supplementary Figure 10 Selected signal associated with long-term artificial rearing. a-c Manhattan plots by F_{ST}, nuclear diversity ($\theta_{\pi\text{-artificially bred}}/\theta_{\pi\text{-wild}}$) and XP-CLR, respectively. The red dashed lines indicated the top 5% thresholds of values, and the blue dashed lines indicated the top 1% thresholds of values. **d** Go analysis of selected genes. **e** KEGG enrichment of selected genes. Source data underlying Supplementary Figure 10 a-c are provided as a Source Dara file, and source data for d-e can be found in Supplementary Data 10 and 11.

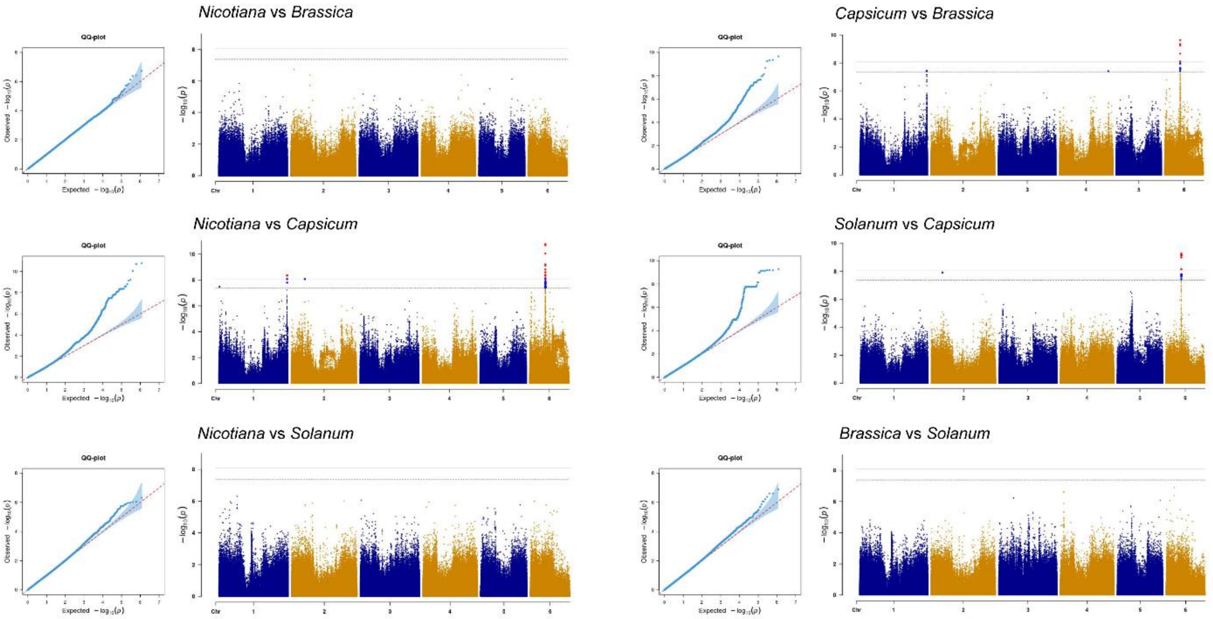

Supplementary Figure 11 Q-Q plots and Manhattan plots of genome-wide association studies (GWAS) compared between different host plant species. The dashed lines and the solid line in Manhattan plots indicated the top 5% and 1% thresholds of values respectively. Source data are provided as a Source Data file.

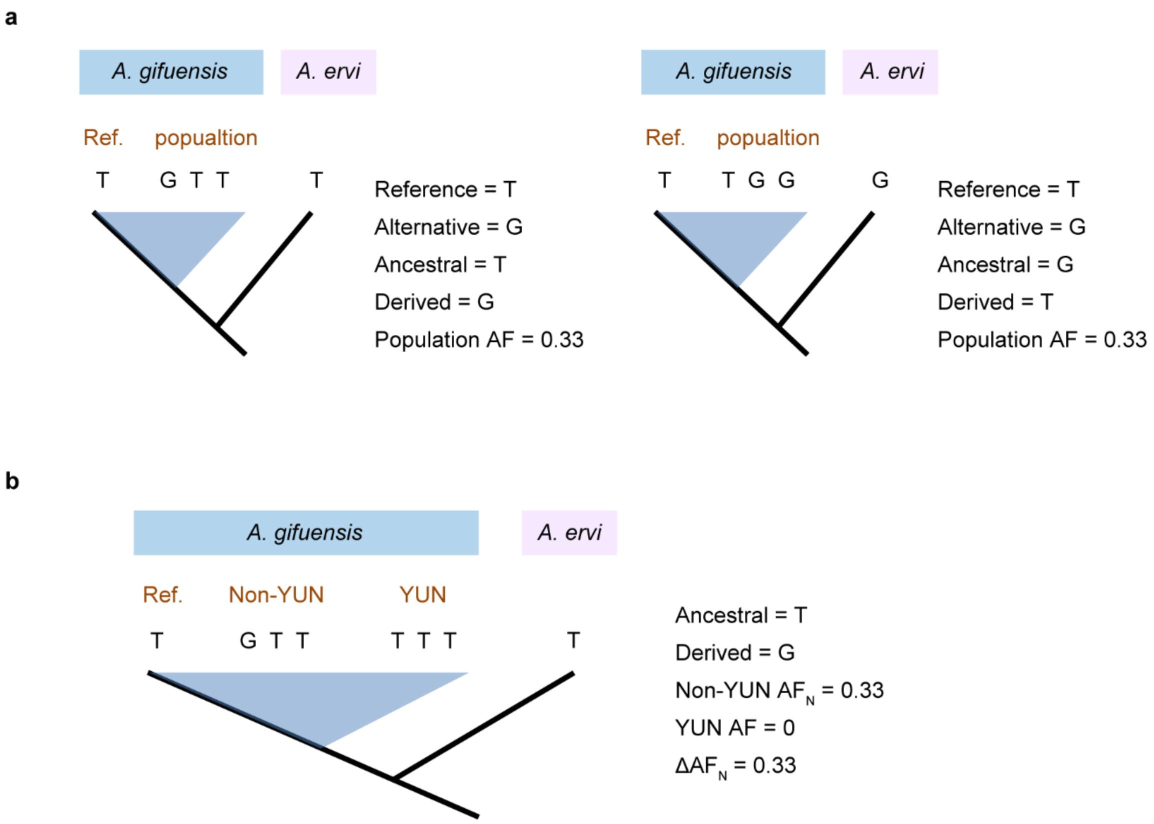

Supplementary Figure 12 Schematic diagram showing how the AF is determined by using an outgroup. a In the correlation tests between SNPs and regional factors, if the reference allele is identical to the *A. ervi* sequence, then the alternative allele is the derived allele. If the alternative allele is identical to the *A. ervi* sequence, then the reference allele is the derived allele. The derived AF is calculated in the *A. gifuensis* population. **b** In the section involving ΔAF, only the non-YUN provinces were investigated. We further required AF = 0 in YUN to ensure that the non-YUN AF_N equals ΔAF, but AFs of the SNPs of interest are exactly identical to those defined in the correlation test.
