# Supplementary material for: Natural selection and genetic diversity maintenance in a parasitic wasp during continuous biological control application
Source: Nat Commun. 2024 Feb 14;15:1379. doi: 10.1038/s41467-024-45631-2 (PMC10866907; doi:10.1038/s41467-024-45631-2)
Supplement: Supplementary file 3 — Description of Additional Supplementary Files [file 41467_2024_45631_MOESM3_ESM.pdf]

Description of Additional Supplementary Files

Title: Supplementary Data 1

Description: Sampling information of *Aphidius gifuensis* wild populations (N). The fourth letter of population ID indicated the type of populations.

Title: Supplementary Data 2

Description: Sampling information of artificially bred (B) and post-release (T) *Aphidius gifuensis* populations. The fourth letter of population ID indicated the type of populations.

Title: Supplementary Data 3

Description: Sequencing and alignment statistics of all wild, artificially bred and post-release individuals.

Title: Supplementary Data 4

Description: Simulation of kinship coefficient between individuals among wild populations.

Title: Supplementary Data 5

Description: Genetic differentiation measured by pairwise-F_{ST} between wild populations.

Title: Supplementary Data 6

Description: The 19 standard bioclimatic variables of wild populations exported from WorldClim2 database with the spatial resolutions of 10 minutes.

Title: Supplementary Data 7

Description: Comparison of population diversity from three groups.

Title: Supplementary Data 8

Description: The linkage disequilibrium decay comparison analysis from each region.

Title: Supplementary Data 9

Description: Statistics of 197 selected genes associated with long-term mass rearing.

Title: Supplementary Data 10

Description: Gene ontology terms of candidate selected genes.

Title: Supplementary Data 11

Description: KEGG catalogs of candidate selected genes.

Title: Supplementary Data 12

Description: Gene introgression between post-release or artificially bred populations and wild populations estimated by ABBA-BABA analysis.

Title: Supplementary Data 13

Description: Candidate genes compared between populations collected from different host plants.

Title: Supplementary Data 14

Description: The accession numbers of individual genomic resequencing data in the Sequence Read Archive (SRA).

Title: Supplementary Data 15

Description: The GenBank accession numbers of individual mitogenomes.
