# Supplementary material for: Natural selection and genetic diversity maintenance in a parasitic wasp during continuous biological control application
Source: Nat Commun. 2024 Feb 14;15:1379. doi: 10.1038/s41467-024-45631-2 (PMC10866907; doi:10.1038/s41467-024-45631-2)
Supplement: Supplementary file 5 — Reporting Summary [file 41467_2024_45631_MOESM5_ESM.pdf]

nature portfolio

Corresponding author(s): Hu Li and Zhonglong Lin

Last updated by author(s): Jan 20, 2024

Reporting Summary

Nature Portfolio wishes to improve the reproducibility of the work that we publish. This form provides structure for consistency and transparency in reporting. For further information on Nature Portfolio policies, see our Editorial Policies and the Editorial Policy Checklist.

Statistics

For all statistical analyses, confirm that the following items are present in the figure legend, table legend, main text, or Methods section.

n/a	Confirmed	
☐	☒	The exact sample size (*n*) for each experimental group/condition, given as a discrete number and unit of measurement
☐	☒	A statement on whether measurements were taken from distinct samples or whether the same sample was measured repeatedly
☐	☒	The statistical test(s) used AND whether they are one- or two-sided<br>*Only common tests should be described solely by name; describe more complex techniques in the Methods section.*
☒	☐	A description of all covariates tested
☐	☒	A description of any assumptions or corrections, such as tests of normality and adjustment for multiple comparisons
☐	☒	A full description of the statistical parameters including central tendency (e.g. means) or other basic estimates (e.g. regression coefficient) AND variation (e.g. standard deviation) or associated estimates of uncertainty (e.g. confidence intervals)
☐	☒	For null hypothesis testing, the test statistic (e.g. *F*, *t*, *r*) with confidence intervals, effect sizes, degrees of freedom and *P* value noted<br>*Give P values as exact values whenever suitable.*
☒	☐	For Bayesian analysis, information on the choice of priors and Markov chain Monte Carlo settings
☒	☐	For hierarchical and complex designs, identification of the appropriate level for tests and full reporting of outcomes
☐	☒	Estimates of effect sizes (e.g. Cohen's *d*, Pearson's *r*), indicating how they were calculated

Our web collection on statistics for biologists contains articles on many of the points above.

Software and code

Policy information about availability of computer code

Data collection	The raster v3.4-10 package in R v3.6.0 was used to download bioclimatic data of WorldClim.
Data analysis	The R package map was used for drawing sample collection map.<br>Fastq 0.21.0 was used for sequencing quality control.<br>BWA-MEM v0.7.12-r1039, SAMtools v1.9, Picard v2.21.6, Genome Analysis Toolkit (GATK) v4.1.9.0, VCFtools v0.1.16 and SnpEff v4.3t were used for population genome resequencing read alignment, variant calling and SNPs annotation.<br>Geneious v11.1.4 was used for mitochondrial genome assembly.<br>KING v2.2.5 was used to estimate kinship coefficient.<br>The populations function of Stacks v2.60 was use to calculate genetic diversity.<br>The R package SNPRelate was used to calculate Weir and Cockerham's estimator of Fst.<br>PLINK v1.90b6.18 was used to prune SNPs for population genetic analyses and assess inbreeding level.<br>ADMIXTURE v1.3.0 and BAPS v6.0 were used to conduct population structure based on SNPs and mitochondrial genome, respectively.<br>RAxML v8.2.12 and IQ-TREE 1.6.5 were used to reconstruct phylogenetic tree.<br>SMC++ v1.15.3 was used to estimate the effective population size (Ne) over time.<br>PopLDdecay was used to measure the degree of linkage disequilibrium (LD).<br>VCFtools v0.1.16 and XP-CLR were used for selective sweeps.<br>The R package clusterProfiler was used for GO enrichment and KEGG pathway analyses.<br>MUSCLE v3.8.31, Pal2Nal and PAML were used to calculate nonsynonymous and synonymous substitution rates (dN, dS) of coding genes.<br>EMMAX was used to analyze the relative kinship of theses populations.

The R package qqman was used for the quantile-quantile (Q-Q) plot and the Manhattan plot.
Dsuite v0.5 was used for the ABBA-BABA analysis.

For manuscripts utilizing custom algorithms or software that are central to the research but not yet described in published literature, software must be made available to editors and reviewers. We strongly encourage code deposition in a community repository (e.g. GitHub). See the Nature Portfolio guidelines for submitting code & software for further information.

Data

Policy information about availability of data

All manuscripts must include a data availability statement. This statement should provide the following information, where applicable:

- Accession codes, unique identifiers, or web links for publicly available datasets
- A description of any restrictions on data availability
- For clinical datasets or third party data, please ensure that the statement adheres to our policy

The raw sequencing data from this study can be found in the Sequence Read Archive (SRA) under Bioproject accession number PRJNA852353, with the accession number of SRR19960681–SRR19961222. All mitogenomes have been deposited in GenBank under the accession numbers OP002317–OP002856. Reference genome of Aphidius gifuensis (GenBank: GCA_014905175.1, https://www.ncbi.nlm.nih.gov/datasets/genome/GCF_014905175.1/) and its mitogenome (GenBank: MT264907.1, https://www.ncbi.nlm.nih.gov/nuccore/MT264907.1/) can be found in NCBI. Source data are provided with this paper.

Research involving human participants, their data, or biological material

Policy information about studies with human participants or human data. See also policy information about sex, gender (identity/presentation), and sexual orientation and race, ethnicity and racism.

Reporting on sex and gender	n/a
Reporting on race, ethnicity, or other socially relevant groupings	n/a
Population characteristics	n/a
Recruitment	n/a
Ethics oversight	n/a

Note that full information on the approval of the study protocol must also be provided in the manuscript.

Field-specific reporting

Please select the one below that is the best fit for your research. If you are not sure, read the appropriate sections before making your selection.

☐ Life sciences ☐ Behavioural & social sciences ☒ Ecological, evolutionary & environmental sciences

For a reference copy of the document with all sections, see nature.com/documents/nr-reporting-summary-flat.pdf

Ecological, evolutionary & environmental sciences study design

All studies must disclose on these points even when the disclosure is negative.

Study description	Population genetic and evolutionary history study of a parasitic wasp Aphidius gifuensis, and assessments on effects of mass rearing and large-scale biocontrol release by comparative population genomic analyses.
Research sample	542 Aphidius gifuensis individuals were collected across China for whole-genome sequencing, including 265 individuals sampled in the wild across their natural range, 160 individuals from artificially bred populations, and 117 individuals from experimental tobacco fields following release (post-release populations) (Supplementary Data 1 and Data 2). Wild populations were used for analyzing Population genetic and evolutionary history, while artificially bred and post-release populations were used to compared with wilds to assess the influences of large-scale biocontrol release on genomic variances.
Sampling strategy	We sequenced at least 5-10 individuals per population, which were limited by the amount of available samples in the fields and the budget to whole-genome sequencing.
Data collection	We collected Aphidius gifuensis adults from mummified Myzus persicae. Field samples (including wilds and individuals from experimental tobacco fields following release) were collected by Bingyan Li, Zengbei Feng, Yunfei Wu, Zhuo Chen, Qian Zhao, Qiaoqiao Liu and Xinzhi Liu., and the location informations of each population were recorded with a GPS tracker in the field. Artificially bred populations were collected from laboratory which reared on Myzus persicae in local Biological Control for Tobacco Diseases and Insect Pests Engineering Research Center across China. Whole-genome sequencing data were collected using Illumina NovaSeq sequencing platform.
Timing and spatial scale	Field and artificially bred Aphidius gifuensis populations were collected from 26 province across China. Population samples were

	collected during the summer when the parasitoid wasp was actively reproducing.
Data exclusions	Sample CQWLN07 with low coverage sequencing and twenty-eight individuals from the wild populations that identified as full- or half-siblings of other individuals (Supplementary Data 4) were excluded.
Reproducibility	Replicating our study by resequencing individuals and reanalysing would be cost-prohibitive.
Randomization	Samples were grouped based on sampling location.
Blinding	Investigators were not blinded to the identity codes of samples. Analyses of genomic data were performed using standardized and automated pipelines of all available samples.

Did the study involve field work? ☐ Yes ☒ No

Reporting for specific materials, systems and methods

We require information from authors about some types of materials, experimental systems and methods used in many studies. Here, indicate whether each material, system or method listed is relevant to your study. If you are not sure if a list item applies to your research, read the appropriate section before selecting a response.

Materials & experimental systems

n/a	Involved in the study
☒	☐ Antibodies
☒	☐ Eukaryotic cell lines
☒	☐ Palaeontology and archaeology
☐	☒ Animals and other organisms
☒	☐ Clinical data
☒	☐ Dual use research of concern
☒	☐ Plants

Methods

n/a	Involved in the study
☒	☐ ChIP-seq
☒	☐ Flow cytometry
☒	☐ MRI-based neuroimaging

Animals and other research organisms

Policy information about studies involving animals; ARRIVE guidelines recommended for reporting animal research, and Sex and Gender in Research

Laboratory animals	We firstly collected mummified Myzus persicae in the laboratory. Female adults were subsequently collected from mummified Myzus persicae after emergence, which were used for genome resequencing.
Wild animals	We collected mummified Myzus persicae from wild, and reared in laboratory at room temperature (~25 ℃) until adults emerging from mummified aphids. Female adults were collected and preserved in anhydrous ethanol.
Reporting on sex	Only female adults were used in this study, because of the unique haplodiploid sex determination in Hymenoptera that unfertilized eggs produce haploid males and fertilized eggs produce diploid females.
Field-collected samples	Mummified Myzus persicae from experimental tobacco fields following release were collected and returned to laborary. Mummified Myzus persicae were reared in laboratory at room temperature (~25 ℃) until adults emerging. Female adults were used for genome resequencing.
Ethics oversight	No ethical approval was required in this case.

Note that full information on the approval of the study protocol must also be provided in the manuscript.
